# Supplementary figures and images for: Species-Specific Discrimination of Insect Meals for Aquafeeds by Direct Comparison of Tandem Mass Spectra
Source: Animals (Basel). 2019 May 7;9(5):222. doi: 10.3390/ani9050222 (PMC6562778; doi:10.3390/ani9050222)

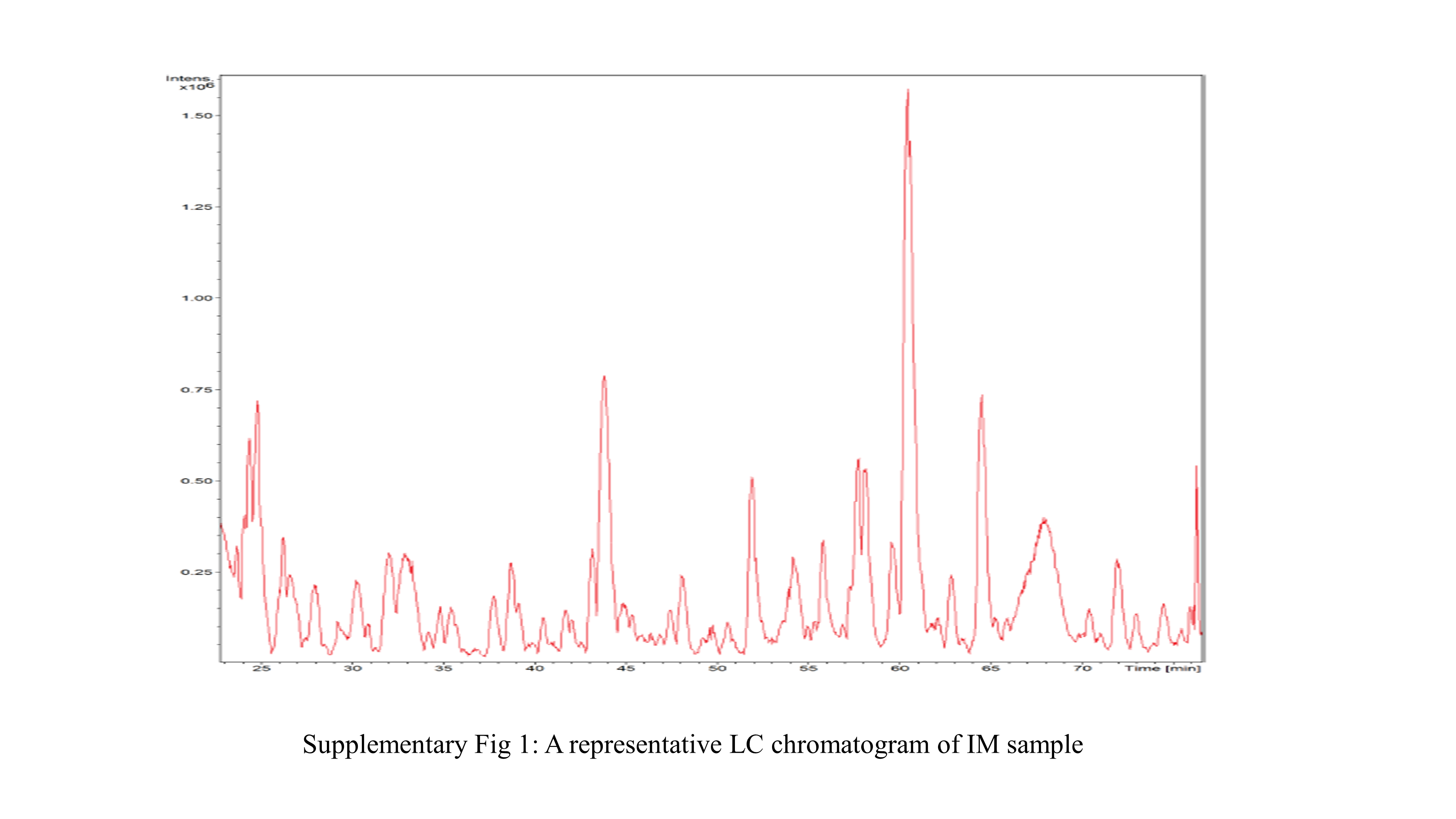

Supplement: Supplementary file 1 [file animals-09-00222-s001.zip › Suppl/Figure S1.tif]
